# Bullying and truancy amongst school-going adolescents in Timor-Leste: results from the 2015 global school-based health survey

**DOI:** 10.1016/j.heliyon.2022.e08797

**Published:** 2022-01-19

**Authors:** Derrick Nyantakyi Owusu, Kenneth Owusu Ansah, Nutifafa Eugene Yaw Dey, Henry Ofori Duah, Pascal Agbadi

**Affiliations:** a Research Department, FOCOS Orthopaedic Hospital, Accra, Ghana; b Department of Psychology, University of Ghana, P.O. BOX LG 84, Legon, Ghana; c Department of Nursing, College of Health Sciences, Kwame Nkrumah University of Science and Technology, PMB, Kumasi, Ghana; d Department of Sociology and Social Policy, Lingnan University, 8 Castle Peak Road, Tuen Mun, Hong Kong

**Keywords:** Bullying, Truancy, School-going adolescents, Timor-leste, Logistic regression

## Abstract

**Introduction:**

Studies in Southeast Asia found that bullying commonly occurred among students, and it has a detrimental impact on their school attendance. However, there is a paucity of literature in Timor-Leste on the association between bullying and truancy. Therefore, this study examined the association between bullying and truancy among Timor-Leste school-going adolescents.

**Methods:**

We used the 2015 Timor-Leste Global School-based Student Health Survey (GSHS) dataset to examine our hypothesis in logistic regression models for both full adolescents (*N* = 3609) and gender stratified samples. The models further controlled for other sociodemographic variables. Statistical significance was pegged at *p* ≤ 0.05, and the analyses were performed in Stata version 14.

**Results:**

About 28% [95% CI:25.7, 30.8] and 36% [95% CI:33.5, 39.5] of school-going adolescents had experienced bullying and truancy, respectively. In-school adolescents who were bullied were more likely to be truant in school even after controlling for the effects of sex, age, grade in school, food insecurity, current substance use, number of friends, colleague support, and parental involvement. This relationship remained significant in the full and gender stratified models. Additionally, school-going adolescents who were currently using substances were truant. Males who were in a physical fight while females who were physically attacked were more likely to be truant.

**Conclusion:**

The study showed that bullying was related to truancy among school-going adolescents in Timor-Leste. Implementation of interventions such as Project START (Stop Truancy and Recommend Treatment) to curtail the incidence of bullying, regulation of current substance use, creating an enabling environment to reduce physical fights, and attacks will significantly reduce the rate of truancy among school-going adolescents in Timor-Leste.

## 1 Introduction

Bullying is a prevalent violent behaviour among children and school-going adolescents (Patton et al., 2017). Literature posits that victims of bullying are likely to experience serious mental and physical health challenges (Gastic, 2008; Karatas and Ozturk, 2011; Nawi et al., 2017). Bullying is defined as an aggressive and intentional act of violence either verbal or physical meted out by a group or individuals repeatedly against a victim who cannot easily defend him or herself (Karatas and Ozturk, 2011). Although this definition has been debated, most researchers concur that bullying involves an intent to harm or demean a defenseless victim (Nawi et al., 2017; Neupane et al., 2020). Variations exist in the rates of bullying victimization across countries. These rates range from 5% to 70% among school-going adolescents (Lund and Ross, 2017). Reasons for these variations are not entirely lucid; however, they can be attributed to cultural factors and social inequalities. School-going adolescents living in regions with wide socioeconomic disparities are found to be at a higher risk of being bullied. A survey conducted in the United States shows that one out of every five students reports being bullied, with males experiencing more bullying than females (Seldin and Yanez, 2019). In Malaysia, another report showed that male adolescents were more likely to be victims of bullying as compared to their female counterparts (Tan et al., 2019). Male adolescents most often experience physical forms of bullying rather than verbal and relational (Seldin and Yanez, 2019; Tan et al., 2019). Some longitudinal studies have shown that the noxious effects of bullying on school-going adolescents could extend through into the adult life of victims (Fullchange and Furlong, 2016; Hemphill et al., 2011; Ttofi et al., 2012). A significant association exists between exposure to bullying and short and long-term consequences such as depression, anxiety, loneliness, suicidal ideations, truancy, lower academic achievements, and dropping out of school (Neupane et al., 2020; Pengpid and Peltzer, 2019a). Bullying also affects the frequency of attending school among in-school adolescents, and truancy can have other adverse outcomes such as poor academic performance (Gastic, 2008; Karatas and Ozturk, 2011; Holt et al., 2013; Neupane et al., 2020; Henry and Yelkpieri, 2017).

The evidence on the association between bullying and truancy is mixed. Whereas studies have established that bullying leads to truancy among in-school adolescents (Dunne et al., 2013; Grinshteyn and Tony Yang, 2017), a few studies indicated no significant association (Attwood and Croll, 2006; Onyeaka et al., 2020; Wilson et al., 2012). These mixed results have been attributed to adolescents’ social environments, different students' adaptation mechanisms to bullying, peer and parent involvement, and the existence of social support structures for students who are bullied (Havik et al., 2015; Kolbert et al., 2014). In South Asia, only a few studies have concluded that bullying was associated with truancy among in-school adolescents in Indonesia, Malaysia, Brunei Darussalam, Myanmar, Philippines, Thailand, and Vietnam (Pengpid and Peltzer, 2017; Smith and Toda, 2016). In the same vein, bullying has been found to have a significant association with truancy among in-school adolescents in Ghana, Zambia, and Mexico (Afful-Broni and Sekyi, 2014; Ruiz-Ramirez et al., 2018; Muula et al., 2012). Given these findings, it is tempting to believe that similar relationships will exist in Timor-Leste, but this cannot be assumed without an empirical investigation.

Given the dearth of information on the effect of bullying on truancy in Timor-Leste, the current study examines this association using a publicly available and nationally representative dataset on in-school adolescents. It further adjusts for the influencing effects of sex, age, grade in school, food insecurity, current substance use, number of friends, colleague support, and parental involvement, variables that have been considered in similar studies and reported to modify the relationship between bullying and truancy (Havik et al., 2015; Kolbert et al., 2014; Pengpid & Peltzer, 2017, 2019b). We hypothesized that bullying would increase the incidence of truancy among Timor-Leste adolescents in school. Building a better understanding of the link between bullying and truancy will help in the development of evidence-based violent behaviour and truancy reduction interventions in Timor-Leste. These interventions are critical in maintaining a safe environment, improving student health, and removing barriers to learning success (Sittichai et al., 2018). It is argued that research investigating gender differences about a topic is key to strengthening intervention implementation (Tannenbaum et al., 2016). Therefore, we additionally examined the link between bullying and truancy separately for male and female adolescents allowing for a more nuanced interrogation.

## 2 Methods

### 2.1 Data source and study design

We used the 2015 Timor-Leste Global school-based student health survey (GSHS), a cross-sectional secondary dataset for this study (WHO, 2017). The GSHS is a nationally representative school-based survey carried out amongst students aged 13–17 years in Low-and-middle income countries around the world. It was initially developed by the World Health Organization (WHO) in 2003. It was developed in partnership with United Nations UNICEF, UNESCO, and UNAIDS with the Centre for Disease Control and Prevention (CDC) providing technical support. The GSHS consists of a wide set of variables including demographics, alcohol and drug use, sexual behaviours that contribute to HIV infection, sexually transmitted infections, unintended pregnancy, tobacco use, violence and unintentional injury, dietary behaviours, hygiene, mental health, and physical activity.

### 2.2 Study setting

Timor-Leste serves as the study's setting. Timor-leste is a lower-middle-income East Asian country with a population of approximately 1.2 million individuals at the time of the 2015 GSHS. It is bordered on the southeast by the Timor Sea, on the north, northwest, and southwest by Wetar Strait, Ombai Strait, and Western Timor (World Bank, 2021).

### 2.3 Sampling and data collection procedure

School-going students aged 13–17 in Class 7–11 from Timor-Leste were included in the GSHS. About 4691 students were eligible for data collection; however, a total of 3,704 students completed the survey. Data was collected from students using a computer scannable answer sheet. It was distributed by trained staff during one standard class period. The survey employed a two-stage cluster sample design to generate representative data of all students in Class 7–11. At the first stage, schools were selected reflecting the probability proportion of the entire enrolment size in the country. Thirty-eight (38) schools were randomly sampled at this stage. At the second stage, classes were randomly chosen with a random start from the sample schools, and all students in these classes qualified to participate. The data collection response rates were 100% school response, 79% student response, and 79% overall data collection response.

### 2.4 Sample size

The sample size of students recruited for this study was 3704. Data of 95 students were dropped due to missing values in the outcome variables. A total of 3609 students were included as the final analytical sample.

## 3 Measures

### 3.1 Predictor variable

The main predictor variable used for this study was bullying. It was measured using this single-item question: “During the past 30 days, on how many days were you bullied?” Students had to indicate their responses to this question on a 7-point response scale namely, “0 days”, “1 or 2 days”, “3–5 days”, “6–9 days”, “10–19 days”, “20–29 days” and “All 30 days”. The GSHS generated a binary variable from this question to capture the percentage of participants who were bullied versus those who were not; that is, “Yes” (1) and “No” (0) respectively (WHO, 2017). It was created by combining participants’ responses on “0 days” into “No” and the remaining response options into “Yes” (WHO, 2017). It is this binary variable we used for our analyses.

### 3.2 Outcome variable

The outcome variable considered in this study was truancy. This variable was also captured in the dataset by a single item question namely, “During the past 30 days, on how many days did you miss classes or school without permission?”. Students responded to the truancy question with “0 days”, “1 or 2 days”, “3–5 days”, “6–9 days”, and “10 to more days”. As described for the predictor variable, a binary variable was also created for the truancy variable by GSHS (WHO, 2017). For this created binary variable, “0 days” were recoded as “No” and the other response options as “Yes” (WHO, 2017). We used the binary variable for this study.

### 3.3 Control variables

We selected control variables per their availability in the dataset and recommendations of previous school-based research (Seidu, 2019; Siziya et al., 2008). Sex, age, grade in school, food insecurity, current substance use, number of friends, colleague support, and parental involvement were the variables selected. The details of the control variables, corresponding survey questions, and the coding used for the statistical analysis are presented in Table 1.

**Table 1:** Variables, survey questions and coding.

Study Variables	Survey questions and coding
**Truancy**	During the past 30 days, on how many days did you miss classes or school without permission?
No	(1) 0 days
Yes	(2) 1 or 2 days/3 to 5 days/6 to 9 days/10 to more days
**Bullying**	During the past 30 days, on how many days were you bullied?
No	(1) 0 days
Yes	(2) 1 or 2 days/3 to 5 days/6 to 9 days/10 to 19 days/20 to 29 days/All 30 days
**Age**	How old are you?
11–15yrs	(0) 11 years old or younger-15 years old
16 + years	(1) 16 years old -18 years old or older
**Sex**	What is your sex?
Male	(1) Male
Female	(2) Female
**Grade in school**	In what grade are you?
Class 7–9, EBC.3 CICLO	(0) Class 7 (EBC. 3 Ciclo)/Class 8 (EBC. 3 Ciclo)/Class 9 (EBC. 3 Ciclo)
Class 10–12 (ES)	(1) Class 10 (ES)/Class 11 (ES)/Class 12 (ES)
**Number close friends**	How many close friends do you have?
0	(1) 0
1	(2) 1
2	(3) 2
3 or more	(4) 3+
**Food insecurity**	During the past 30 days, how often did you go hungry because there was not enough food in your home?
No	(0) Never/Rarely/Sometimes
Yes	(1) Most of the time/Always
**Current substance use**	During the past 30 days, on how many days did you have at least one drink containing alcohol?
	(0) 0 days
	(1) 1 or 2 days/1 to 2 days/3 to 5 days/6 to 9 days/10 to 19 days/20 to 29 days/All 30 days
	During the past 30 days, on how many days did you smoke cigarettes?
	(0) 0 days
	(1) 1 or 2 days/1 to 2 days/3 to 5 days/6 to 9 days/10 to 19 days/20 to 29 days/All 30 days
	During the past 30 days, how many times have you used marijuana (also called Ganja)?
	(0) 0 days
	(1) 1 or 2 times/3 to 9 times/10 to 19 times/20 or more times
	During the past 30 days, on how many days did you use any tobacco products other than cigarettes, such as Joker, LA, Gudang garam, Sigaru 23, Surya, snuff, chewing tobacco, or betel?
No	(0) 0 days
Yes	(1) 1 or 2 days/1 to 2 days/3 to 5 days/6 to 9 days/10 to 19 days/20 to 29 days/All 30 days
**Physical fight**	During the past 12 months, how many times were you in a physical fight?
No	(1) 0 days
Yes	(2) 1 time/2 or 3 times/4 or 5 times/6 or 7 times/8 or 9 times/10 or 11 times/12 or more times
**Physical attack**	During the past 12 months, how many times were you physically attacked?
No	(0) 0 days
Yes	(1) 1 time/2 or 3 times/4 or 5 times/6 or 7 times/8 or 9 times/10 or 11 times/12 or more times
**Loneliness**	During the past 12 months, how often have you felt lonely?
No	(0) Never/Rarely/Sometimes
Yes	(1) Most of the time/Always
**Anxiety**	During the past 12 months, how often have you been so worried about something that you could not sleep at night?
No	(0) Never/Rarely/Sometimes
Yes	(1) Most of the time/Always
**Helpful colleagues**	During the past 30 days, how often were most of the students in your school kind and helpful?
No	(0) Never/Rarely/Sometimes
Yes	(1) Most of the time/Always
**Parental supervision**	During the past 30 days, how often did your parents or guardians check to see if your homework was done?
No	(0) Never/Rarely/Sometimes
Yes	(1) Most of the time/Always
**Parental connectedness**	During the past 30 days, how often did your parents or guardians understand your problems and worries?
No	(0) Never/Rarely/Sometimes
Yes	(1) Most of the time/Always
**Parental bonding**	During the past 30 days, how often did your parents or guardians really know what you were doing with your free time?
No	(0) Never/Rarely/Sometimes
Yes	(1) Most of the time/Always

### 3.4 Ethics and data accessibility

Ethical approval for the survey was granted by the World Health Organization's Ethical Committee. In addition to this, ethical clearance was sought from and granted by the ministries of education and/or health in each participating country. The WHO has made the datasets for Timor-Leste freely available and accessible to the public on their website https://www.cdc.gov/gshs/countries/seasian/timor_leste.htm.

### 3.5 Data analysis

Data analysis was conducted in Stata version 14. Survey weights were applied, and afterward, univariable analysis was performed. Before beginning the main study analysis (i.e., bivariable and multivariable analysis), we accounted for the complex sampling design embedded in the dataset to control potential analytic errors and allow proper inferences (see West et al., 2016). This was possible by activating the complex survey command “svyset” in Stata, adjusting for clusters, stratification, and sample weights. When this was done, we carried out the bivariable analysis in chi-square estimating the relationship between bullying, covariates, and truancy. This was done on the full sample and separately on the gender stratified samples. Next, we performed the multivariable analysis in logistic regression by entering the “logistic” command to predict bullying on truancy without the control variables. We then performed the same analysis but this time included the control variables. The multivariable analysis as seen in Table 5 was first performed on the full sample and subsequently on the gender stratified samples. Statistical significance was set at *p* ≤ 0.05 since this level of significance is widely acceptable for hypothesis testing and additionally helps avoid type one error (Dahiru, 2008; Grabowski, 2016). Both odd ratios (OR) estimates and adjusted odds ratios (AOR) estimates are reported in Table 5.

## 4 Results

### 4.1 Intercorrelation matrix

The Inter-correlation matrix which indicates the relationships among the study variables was performed using the Pearson Product Moment Correlation. From Table 2, a significant relationship between bullying and truancy was established. Tabachnick and Fidell (2012) opined that in any regression analysis, one must ensure linearity between the various independent variables, which was met in this study. The correlation coefficients between the various independent variables to be used in the regression analysis were also below 0.70, not violating the multicollinearity ruleas suggested by Tabachnick and Fidell (2012).

**Table 2:** Correlation matrix of all study variables.

Study variables	1	2	3	4	5	6	7	8	9	10	11	12	13	14	15	16
1. Truancy	—															
2. Bullying	0.20∗∗∗	—														
3. Age	0.05∗∗	-0.06∗∗	—													
4. Sex	-0.11∗∗∗	-0.12∗∗∗	-0.06∗∗∗	—												
5. Grade in school	0.03	-0.04∗	0.53∗∗∗	0.01	—											
6. Number close friends	-0.05∗∗	-0.00	0.04∗	-0.06∗∗	0.04∗	—										
7. Food insecurity	0.06∗∗∗	0.09∗∗∗	0.01	-0.02	0.01	-0.05∗∗	—									
8. Substance use	0.28∗∗∗	0.27∗∗∗	0.11∗∗∗	-0.30∗∗∗	0.10∗∗∗	-0.04∗	0.07∗∗∗	—								
9. Physical fight	0.16∗∗∗	0.38∗∗∗	-0.10∗∗∗	-0.12∗∗∗	-0.10∗∗∗	-0.03	0.08∗∗∗	0.23∗∗∗	—							
10. Physical attack	0.15∗∗∗	0.30∗∗∗	-0.04∗	-0.04∗	-0.03	-0.02	0.07∗∗∗	0.13∗∗∗	0.38∗∗∗	—						
11. Loneliness	0.05∗∗	0.09∗∗∗	0.07∗∗∗	-0.04∗	0.05∗	-0.05∗∗	0.12∗∗∗	0.07∗∗∗	0.06∗∗∗	0.05∗∗	—					
12. Anxiety	0.07∗∗∗	0.11∗∗∗	0.08∗∗∗	-0.03	0.07∗∗∗	-0.01	0.11∗∗∗	0.11∗∗∗	0.10∗∗∗	0.03∗	0.21∗∗∗	—				
13. Helpful colleagues	-0.00	-0.01	0.04∗	0.05∗∗	0.04∗	0.08∗∗∗	0.01	-0.01	-0.00	-0.02	0.06∗∗∗	0.06∗∗∗	—			
14. Parental supervision	-0.02	-0.05∗∗	0.00	0.02	-0.01	0.05∗∗	0.03	-0.04∗	-0.02	-0.06∗∗∗	0.06∗∗∗	0.05∗∗	0.22∗∗∗	—		
15. Parental connectedness	0.04∗∗	0.06∗∗∗	0.06∗∗∗	0.01	0.09∗∗∗	-0.00	0.04∗	0.03	0.04∗	0.00	0.06∗∗∗	0.09∗∗∗	0.15∗∗∗	0.16∗∗∗	—	
16. Parental bonding	0.01	0.03	0.07∗∗∗	0.03	0.10∗∗∗	0.05∗∗	0.00	0.03	-0.01	-0.01	0.07∗∗∗	0.05∗∗	0.18∗∗∗	0.20∗∗∗	0.27∗∗∗	—

Note. ∗*p* < 05; ∗∗*p* < 0.01; ∗∗∗*p* < 0.001.

### 4.2 Participant's characteristics

Of the total sample (*N* = 3609), about 60% were 16 years old and above whereas 40% were aged 11–15 years (Table 3). The gender ratio was approximately 1:1. About 60% were in Class 7–9 whereas 40% were in Class 10–12 (Table 3). Adolescents from food-insecure households constituted 11.43% of the study sample. Only about 5% had no close friends (Table 3). Approximately 28% reported engaging in a physical fight and 14.71% reported loneliness (Table 3). About 12% reported that they were anxious that they could not sleep whiles 28% reported having helpful friends (Table 3). In terms of parental support, 28.43% of parents assisted their children to complete their homework (Table 3). About 12% of parents understood the problems their children were experiencing and 23% had knowledge about where their children spent their free time (Table 3).

**Table 3:** Socio-demographic characteristics of the study.

Study variables	Total Sample	Males sample	Females sample
	n (%)	n (%)	n (%)
**Truancy**			
No	2307 (63.74)	923 (58.9)	1272 (69.1)
Yes	1302 (36.26)	658 (41.1)	569 (30.9)
**Bullying**			
No	2221 (71.80)	908 (67.02)	1222 (77.71)
Yes	925 (28.20)	477 (32.98)	390 (22.29)
**Age**			
11–15yrs	1713 (40.50)	678 (36.62)	946 (42.71)
16 + years	1828 (59.50)	893 (63.38)	890 (57.29)
**Sex**			
Male	1581 (50.41)		
Female	1841 (49.59)		
**Grade in school**			
Class 7–9, EBC.3 CICLO	2383 (60.25)	1043 (60.38)	1253 (59.68)
Class 10–12 (ES)	1127 (39.75)	520 (39.62)	575 (40.32)
**Number close friends**			
0	165 (4.68)	67 (4.14)	86 (4.87)
1	360 (9.55)	133 (8.15)	199 (10.4)
2	563 (14.65)	205 (12.5)	322 (16.24)
3 or more	2453 (71.13)	1145 (75.22)	1205 (68349)
**Food insecurity**			
No	3144 (88.57)	1376 (88.24)	1615 (89.4)
Yes	413 (11.43)	190 (11.76)	195 (10.6)
**Current substance use**			
No	2364 (64.38)	808 (50.35)	1450 (79.6)
Yes	1244 (35.62)	772 (49.65)	391 (20.4)
**Physical fight**			
No	2532 (71.89)	1032 (67.13)	1381 (77.53)
Yes	1056 (28.11)	539 (32.87)	450 (22.47)
**Physical attack**			
No	2205 (62.32)	933 (60.68)	1157 (64.1)
Yes	1388 (37.68)	642 (39.32)	676 (35.9)
**Loneliness**			
No	3015 (85.29)	1299 (84.06)	1579 (87.16)
Yes	499 (14.71)	240 (15.94)	221 (12.84)
**Anxiety**			
No	3167 (87.63)	1373 (87.09)	1647 (89.12)
Yes	419 (12.37)	199 (12.91)	186 (10.88)
**Helpful colleagues**			
No	2569 (72.18)	1161 (74.19)	1272 (69.67)
Yes	951 (27.82)	384 (25.81)	525 (30.33)
**Parental supervision**			
No	2562 (71.57)	1135 (72.5)	1294 (70.71)
Yes	1004 (28.43)	427 (27.7)	526 (29.29)
**Parental connectedness**			
No	3169 (88.24)	1394 (88.45)	1619 (88.15)
Yes	392 (11.76)	167 (11.55)	201 (11.85)
**Parental bonding**			
No	2731 (76.62)	1194 (77.71)	1393 (75.25)
Yes	23.38 (23.38)	336 (22.29)	417 (24.75)

### 4.3 Prevalence of bullying and truancy

**Figure 1:**
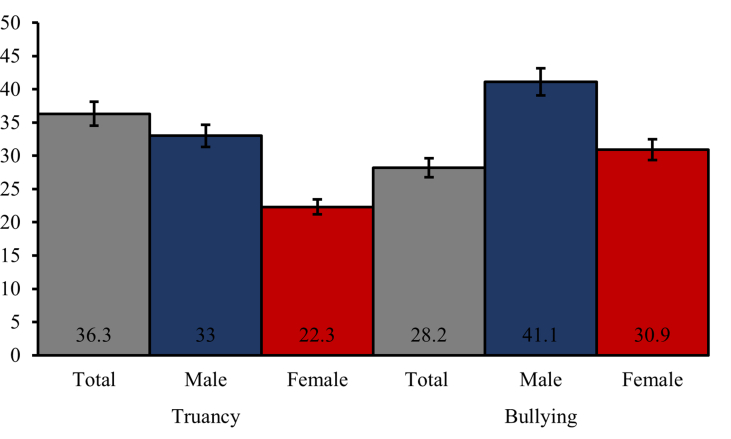
Prevalence rates of bullying and truancy of Timor-Leste adolescents for total and gender-stratified sample.

As seen in Figure 1, the total prevalence rate of truancy was approximately 36.3% [95% CI: 33.5, 39.5] while that of bullying was 28.2% [95% CI:25.7, 30.8]. With regards to gender differences, it was observed that more males experienced bullying victimization [33%; 95% CI: 29.5, 36.7] and truancy [41.1%; 95% CI: 37.9, 44.4] compared females.

### 4.4 Summary of chi-square test for the association between bullying and truancy

Chi-square test for independence was used to assess the association between truancy from school and bullying as well as the covariates (see Table 4). The following variables were found to be significantly associated with truancy in the full sample: bullying, gender, age, food insecurity, current substance use, physical fights, physical attacks, loneliness, and anxiety. Some significant relationships were also revealed in the gender stratified model. In the male sample, bullying, current substance use, physical fight, physically attacked, and parental supervision were significantly related to truancy. For that of the female sample, truancy was related to bullying, food insecurity, current substance use, physical fight, physically attacked, anxiety, and parental connectedness.

**Table 4:** Chi-Square examining the relationship between bullying, covariates, and truancy stratified by gender.

Study Variables	Truancy (Full sample)		Truancy (Male)		Truancy (Female)	
	No	Yes	No	Yes	No	Yes
**Bullying**	*p* < 0.001		*p* < 0.001		*p* < 0.001	
No	71.18%	28.82%	67.58%	32.42%	74.15%	25.85%
Yes	49.84%	50.16%	44.41%	55.59%	59.56%	40.44%
**Gender**	*p* = 0.0002					
Male	58.90%	41.10%				
Female	69.10 %	30.90%				
**Age**	*p* = 0.048		*p* = 0.058		*p* = 0.197	
11–15yrs	66.95%	33.05%	63.64%	36.36%	70.94%	29.06%
16 + years	61.76%	38.24%	56.30%	43.70%	67.76%	32.24%
**Grade in school**	*p* = 0.150		*p* = 0.348		*p* = 0.360	
Class 7-9	65.13%	34.87%	60.45%	39.55%	70.35%	29.65%
Class 10-12	62.12 %	37.88%	57.34%	42.66%	67.59%	32.41%
**Number close friends**	*p* = 0.077		*p* = 0.260		*p* = 0.246	
0	55.22%	44.78%	48.66%	51.34%	62.30%	37.70%
1	58.29%	41.71%	52.82%	47.18%	64.29%	35.71%
2	64.46%	35.54%	60.76%	39.24%	67.48%	32.52%
3 or more	65.03%	34.97%	59.78%	40.22%	70.90%	29.10%
**Food insecurity**	*p* = 0.001		*p* = 0.092		*p* = 0.007	
No	64.76%	35.24%	59.47%	40.53%	70.39%	29.61%
Yes	55.95%	44.05%	53.85%	46.15%	58.86%	41.14%
**Current Substance use**	*p* < 0.001		*p* < 0.001		*p* < 0.001	
No	73.69%	26.31%	72.99%	27.01%	74.30%	25.70%
Yes	45.81%	54.19%	44.68%	55.32%	48.79%	51.21%
**Physical fight**	*p* < 0.001		*p* < 0.001		*p* = 0.008	
No	68.62%	31.38%	65.15%	34.85%	71.76%	28.24%
Yes	51.34%	48.66%	46.24%	53.76%	60.04%	39.96%
**Physically attack**	*p* < 0.001		*p* < 0.001		*p* < 0.001	
No	69.46%	30.54%	65.29%	34.71%	73.94%	26.06%
Yes	54.30%	45.70%	48.93%	51.07%	60.63%	39.37%
**Loneliness**	*p* = 0.015		*p* = 0.337		*p* = 0.139	
No	64.89%	35.11%	60.06%	39.94%	70.00%	30.00%
Yes	58.68%	41.32%	55.16%	44.84%	64.02%	35.98%
**Anxiety**	*p* = 0.011		*p* = 0.101		*p* = 0.007	
No	65.18%	34.82%	60.12%	39.88%	70.36%	29.64%
Yes	54.68%	45.32%	51.19%	48.81%	59.96%	40.04%
**Helpful colleagues**	*p* = 0.885		*p* = 0.761		*p* = 0.641	
No	64.28%	35.72%	59.67%	40.33%	69.28%	30.72%
Yes	64.56%	35.44%	58.78%	41.22%	70.38%	29.62%
**Parental supervision**	*p* = 0.275		*p* = 0.043		*p* = 0.435	
No	63.37%	36.63%	57.69%	42.31%	69.75%	30.25%
Yes	65.49%	34.51%	63.50%	36.50%	67.89%	32.11%
**Parental connectedness**	*p* = 0.053		*p* = 0.382		*p* = 0.040	
No	64.74%	35.26%	59.80%	40.20%	70.15%	29.85%
Yes	58.07%	41.93%	55.29%	44.71%	60.62%	39.38%
**Parental Bonding**	*p* = 0.740		*p* = 0.814		*p* = 0.628	
No	64.22%	35.78%	59.46%	40.54%	69.47%	30.53%
Yes	63.41%	36.59%	58.64%	41.36%	68.17%	31.83%

### 4.5 Summary of logistic regression analysis for the association between bullying and truancy

To test the hypothesis of the association between bullying and truancy, a logistic regression analysis was performed on the full adolescent sample and gender stratified samples. In the full sample model, the experience of bullying was associated with 2.49 times greater odds of truancy among school children in the crude analysis [OR = 2.49, 95% CI:2.05, 3.02] (Table 5). After adjusting for gender, age, grade, food insecurity, current substance use, number of close friends, physical fights, anxiety, having helpful colleagues, and parental support variables, adolescents who experience bullying were 1.63 times likely to be truant in school [AOR = 1.63, 95% CI:1.37, 1.94] (see Table 5). This finding supports our hypothesis that bullying increases the likelihood of truancy among in-school adolescents in Timor-Leste.

**Table 5:** Bivariate and multivariate complex sample logistic regression estimates predicting child bullying on truancy stratified by gender.

Study variables	Full model	Male model	Female model
**Model [OR]**	**[95% CI]**	**[95% CI]**	**[95% CI]**
**Bullying**			
No	1 [ref]	1 [ref]	1 [ref]
Yes	2.49∗∗∗[2.05, 3.02]	2.61∗∗∗[2.07, 3.29]	1.947∗∗∗[1.47, 2.57]
**Model [AOR]**	**[95% CI]**	**[95% CI]**	**[95% CI]**
**Bullying**			
No	1 [ref]	1 [ref]	1 [ref]
Yes	1.63∗∗∗[1.37, 1.94]	1.84∗∗∗[1.47,2.31]	1.45∗∗[1.13,1.86]
**Age**			
11–15 years	1 [ref]	1 [ref]	1 [ref]
16 + years	1.23 [0.88, 1.72]	1.31 [0.76, 2.26]	1.14 [0.81, 1.59]
**Grade in school**			
Class 9-11	1 [ref]	1 [ref]	1 [ref]
Class 10-12	0.98 [0.67, 1.44]	0.92 [0.55, 1.56]	1.05 [0.70, 1.58]
**No close friends**			
0	1 [ref]	1 [ref]	1 [ref]
1	0.73 [0.43, 1.26]	0.70 [0.24, 2.05]	0.76 [0.34,1.70]
2	0.72 [0.43, 1.22]	0.62 [0.31, 1.22]	0.77 [0.35, 1.70]
3 or more	0.69 [0.40, 1.17]	0.64 [0.32, 1.30]	0.71 [0.31, 1.63]
**Food insecurity**			
Secure	1 [ref]	1 [ref]	1 [ref]
Insecure	1.19 [0.86, 1.65]	1.19 [0.86, 1.64]	1.22 [0.76, 1.96]
**Current substance use**			
No	1 [ref]	1 [ref]	1 [ref]
Yes	2.38∗∗∗ [1.84, 3.07]	2.50∗∗∗ [1.77, 3.54]	2.21∗∗∗ [1.64, 2.97]
**Physical fight**			
No	1 [ref]	1 [ref]	1 [ref]
Yes	1.26 [0.99, 1.60]	1.45∗∗ [1.11, 1.90]	1.02 [0.63, 1.64]
**Physically attack**			
No	1 [ref]	1 [ref]	1 [ref]
Yes	1.51∗∗∗ [1.24, 1.85]	1.29 [0.94, 1.77]	1.77∗∗∗ [1.32, 2.37]
**Loneliness**			
No	1 [ref]	1 [ref]	1 [ref]
Yes	0.97 [0.74, 1.26]	0.87 [0.52, 1.45]	1.11 [0.71, 1.73]
**Anxiety**			
No	1 [ref]	1 [ref]	1 [ref]
Yes	1.02 [0.67, 1.56]	0.81 [0.50, 1.31]	1.32 [0.81, 2.14]
**Helpful colleagues**			
No	1 [ref]	1 [ref]	1 [ref]
Yes	0.97 [0.80, 1.16]	1.13 [0.88, 1.47]	0.85 [0.67, 1.07]
**Parental supervision**			
No	1 [ref]	1 [ref]	1 [ref]
Yes	0.99 [0.80, 1.22]	0.84 [0.62, 1.14]	1.17 [0.89, 1.53]
**Parental connectedness**			
No	1 [ref]	1 [ref]	1 [ref]
Yes	1.22 [0.93, 1.60]	1.01 [0.66, 1.52]	1.41 [0.90, 2.21]
**Parental Bonding**			
No	1 [ref]	1 [ref]	1 [ref]
Yes	0.89 [0.70, 1.14]	1.03 [0.72, 1.46]	0.77 [0.53, 1.10]
**Gender**			
Female	1 [ref]		
Male	1.14 [0.84, 1.54]		
**Model details**			
Number of observations	2685	1,226	1,459
Population size (weighted)	62,095	31,038	31,056
Strata	19	19	19
PSU	38	38	38
Design *df*	19	19	19
F statistics	*F* (17, 3) = 281.24, *p* = 0.0003	*F* (16, 4) = 17.76, *p* = 0.0065	*F* (16, 4) = 45.67, *p* = 0.001
McKelvey and Zavoina's *R*^2^	0.770	0.757	0.715

*Note*. 95% confidence intervals in brackets; ∗∗*p* < 0.01, ∗∗∗*p* < 0.001.

Bullying was also associated with truancy in both male and female crude and adjusted models, further providing support for our hypothesis. In the adjusted model, we found that males who experienced bullying were 1.84 times more likely to be truant [AOR = 1.84, 95% CI:1.47, 2.31] which was a bit higher than the 1.45 times found among females [AOR = 1.45, 95% CI:1.13, 1.86]. Some covariates were also significantly related to truancy in all three models. Current substance use was related to a high chance of truancy in all three models. For the full model, adolescents who currently use at least one type of substance were 2.38 times more likely to be truant [AOR = 2.38, 95% CI:1.84, 3.07]. This ratio was slightly different in the gender stratified models. Males who were current users of substances were 2.50 times more likely to be absent from school [AOR = 2.50, 95% CI:1.77, 3.54] while females were 2.21 times more likely [AOR = 2.21, 95% CI:1.64, 2.97]. Another significant association that was found in the full sample model was between being physically attacked and truancy [AOR = 1.51, 95% CI:1.24, 1.85]. However, in the gender stratified model, this relationship stayed significant only for females but not males, implying that females who were physically attacked were 1.77 times more likely to be truant [AOR = 1.77, 95% CI:1.32, 2.37]. Interestingly, males who were in a physical fight had a 1.45 increased likelihood of being truant [AOR = 1.45, 95% CI:1.11, 1.90]. This relationship was unique only for males but not females nor the full sample.

## 5 Discussion

This study mainly investigated the effect of bullying on truancy among school-going adolescents in Timor-Leste. Overall, about 28% of the school-going adolescents reported having experienced bullying which is slightly lower than the prevalence rate of 36% reported in a study that used the GSHS data of Myanmar, Pakistan, and Sri Lanka, other Asian countries with similar socio-cultural backgrounds as Timor-Leste (Murshid, 2017). The overall prevalence of truancy was 36.26%. This burden of truancy is higher compared to GSHS reports from other Asian countries that estimate 18%, 15%, 28% of truancy in Thailand, Vietnam, and Malaysia, respectively (Pengpid and Peltzer, 2017). Males, compared to females, reported more bullying and truancy occurrences, and these findings are consistent with previously published empirical works (Onyeaka et al., 2020, Seidu et al., 2019, Seldin and Yanez, 2019, Tan et al., 2019).

We found that school-going adolescents in Timor-Leste who experienced bullying were more likely to be truant from school in both the full sample and gender stratified models. This group of in-school adolescents may have found the school environment threatening and their coping response may have been to dissociate from the threat, eventually manifesting as truancy. This finding is congruent with other studies that reported bullying as a significant predictor of truancy among school-going adolescents (Seidu, 2019; Tan et al., 2019). The relationship between bullying and truancy may also be mediated by poor mental health outcomes (e.g., anxiety, loneliness, and depression) and decreased academic performance (Abdelaziz and Abu-Snieneh, 2021; Hysing et al., 2019; Vaughn et al., 2013). Put simply, bullying victimization has the potential of negatively affecting the mental health and academic performance of adolescents and this could trigger truant behaviours. In the gender stratified model, we consider some non-competing explanations for the association between bullying and truancy. First, physical and psychological assault can undermine male victims’ sense of masculinity since they may believe they have failed to maintain control and power (Shorey et al., 2012). This reduced sense of masculinity may cause them to miss school to avoid being ridiculed by peers. Because female adolescents pay close attention to their physical appearance (Halim et al., 2018), any form of assault that targets their physical appearance may cause them to develop cognitive biases toward interpreting further social rejection, as well as use strategies such as missing school to avoid further victimization (Zimmer-Gembeck and Nesdale, 2013).

School-going adolescents who use substances were more likely to be truant than those who do not use a substance. This link was also significant in both male and female models. Substance abuse can cause "amotivational syndrome," which is characterized by a lack of interest in achieving one's goals (Lac and Luk, 2018). Students who are heavy drug users frequently miss class because they have little desire to achieve any academic feat. Additionally, substance use largely affects cognitive functioning by impairing judgment, motivation, and decision-making (Morin et al., 2019). Such levels of impairments can potentially subdue adolescents' motivation to go to school. Another plausible reason for this finding could be that adolescents using substances might be clinically addicted and unable to manage their cravings. To alleviate their urges, these addicted adolescents may purposefully miss school to look for illicit drugs outside of school grounds. Our finding agreed with what was found in previous studies (Hunt and Hopko, 2009; Maynard et al., 2017; Onyeaka et al., 2020; Pengpid and Peltzer, 2017, 2019).

The findings further revealed that male adolescents who engaged in physical fights were more likely to be truant. Males are considered to have higher levels of testosterone which increases their aggressive tendencies (Batrinos, 2012). Displaying such aggressive tendencies in school come with some punitive measures from authorities. Hence, belligerent adolescents are more likely to absent themselves from school to avoid punishments meted out to them by the authorities. Males who engage in physical fighting have a high chance of sustaining injuries that require medical attention (Seidu et al., 2019) and because of this, they are likely to miss school just to attend to their wounds. In many social contexts, excessively belligerent adolescents are often avoided by their peers (Watling Neal, 2010). This could mean that males who engage in a fight are frequently excluded by their peers and thus, have fewer friends (Da Silva et al., 2016). This may result in feelings of loneliness which in turn could reinforce the behaviour of absenteeism since they do not have anyone in school to bond with. Our result is consistent with earlier research that has reported the link between adolescents being involved in physical fights and truancy (Onyeaka et al., 2020; Pengpid and Peltzer, 2017; Shaikh et al., 2020).

Finally, female adolescents who were physically attacked were more likely to be truant. Generally, females are considered to have heightened reactivity patterns to danger (McLaughlin et al., 2015). Being on the receiving end of physical abuse is a serious threat to their lives. Females are likely to make efforts by adopting truant behaviours to avoid imminent danger. Also, being physically attacked could be humiliating for female adolescents. They are likely to experience depression and anxiety after being physically attacked (Al-Fayez et al., 2012; Cougle et al., 2010) which could result in truant behaviours. Female adolescents thus may begin to have low drive or energy to engage in their daily academic routines. As such, they would likely have little desire to be in school. Our study shares a similarity with that of Pengpid and Peltzer (2017).

### 5.1 Implications

The results of this study call for a national joint initiative in Timor-Leste by various stakeholders to institute measures aimed at reducing the incidence of truancy among school-going adolescents. That is, a multidimensional approach such as Project START which aims to encourage schools, communities, and courts to work together to reduce truancy, could be institutionalized. The findings of this study could also be used by the Ministry of Education in Timor-Leste to establish a school-learning environment free of threats, bullying, and physical attacks. As such, a school-based intervention mentoring programme such as “Check and Connect” could be used to deploy teachers as mentors for absentees. By implementing these strategies, the government of Timor-Leste will be a step closer to ensuring that all adolescents have access to inclusive and high-quality education and encourage lifelong learning as outlined in SDG 4 (UNESCO, 2017). Our study results also call for the inclusion of trained psychologists at various Timor-Leste schools. These psychologists will offer rehabilitation services for school-going adolescents who are victims of bullying, physical attacks, and other forms of threats in hope of reducing absenteeism. Finally, NGOs interested in or already engaging with school-going adolescents can direct their activities to promote building a friendly school atmosphere and fostering a healthy inter-peer relationship, paying close attention to the gender dynamics revealed in our study.

### 5.2 Strengths and limitations

The major strength of this study has to do with the use of a nationally representative sample with a relatively large sample size which increases generalizability. Our findings also extend the literature on bullying and truancy by stratifying the full sample by gender. However, this study has some limitations that need to be highlighted. The study used a cross-sectional design and as such, inferences between the predictors and truancy could only be considered as associations rather than causal relationships. Another limitation is the use of self-reported data. Although the GSHS was designed to capture quick and anonymized nationwide self-report data, it is possible adolescents may have misreported their experience of bullying and truancy due to recall bias and social desirability responding. Additionally, adolescents who were absent on the day of data collection were not included in the study, limiting the generalizability of the findings. Variations in truancy amongst early adolescence vs. late adolescence have also been reported elsewhere (Maynard et al., 2017). Future studies using data from Timor-Leste could, therefore, look at age differences in the factors relating to truancy.

## 6 Conclusion

Bullying victimization was related to truancy in all models considered in this study: total and male-female samples. Other predictive factors of truancy were current substance use, physical fighting, and physical attacks. We, therefore, propose that intervention attempts to reduce truancy among school-going adolescents in Timor-Leste should be directed through reducing the incidence of bullying, current substance use, physical fights, and attacks in the school environment.

## Declarations

### Author contribution statement

Derrick Nyantakyi Owusu: Contributed reagents, materials, analysis tools or data; Wrote the paper.

Kenneth Owusu Ansah, Nutifafa Eugene Yaw Dey: Conceived and designed the experiments; Analyzed and interpreted the data; Wrote the paper.

Henry Ofori Duah: Conceived and designed the experiments; Contributed reagents, materials, analysis tools or data; Analyzed and interpreted the data;Wrote the paper.

Pascal Agbadi: Conceived and designed the experiments; Analyzed and interpreted the data. Wrote the paper.

### Funding statement

This research did not receive any specific grant from funding agencies in the public, commercial, or not-for-profit sectors.

### Data availability statement

Data associated with this study has been deposited at WHO under the urL: https://www.who.int/ncds/surveillance/gshs/timorleste/en/or https://www.cdc.gov/gshs/countries/seasian/timor_leste.htm.

### Declaration of interests statement

The authors declare no conflict of interest.

### Additional information

No additional information is available for this paper.
